# The Oral–Gut–Brain Axis: From Periodontal Dysbiosis to Neuroinflammation—Mechanistic Pathways, Salivary and Intestinal Biomarkers, and Therapeutic Targets: A Narrative Review

**DOI:** 10.3390/dj14050289

**Published:** 2026-05-11

**Authors:** Caterina Nela Dumitru, Alina Oana Dumitru, Gabriel Valeriu Popa, Teodora Marcu, Maria Ursu, Aurel Nechita, Nicoleta Madalina Matei

**Affiliations:** 1 Research Centre in the Medical-Pharmaceutical Field, Faculty of Medicine and Pharmacy, “Dunărea de Jos” University of Galați, 35 Al. I. Cuza Street, Galați County, 800010 Galați, Romania; 2 “Sf. Cuvioasa Parascheva” Clinical Hospital of Infectious Diseases, Galați County, 800179 Galați, Romania; 3 Pharmaceutic Department, Faculty of Medicine and Pharmacy, “Dunărea de Jos” University of Galati, 800201 Galati, Romania; 4 Faculty of Medicine and Pharmacy, “Dunărea de Jos” University of Galați, 35 Al. I. Cuza Street, Galați County, 800010 Galați, Romania; 5 Dental-Medicine Department, Faculty of Medicine and Pharmacy, “Dunărea de Jos” University of Galati, 800201 Galati, Romania; 6 “Sf. Ioan” Clinical Emergency Pediatric Hospital in Galati, 800487 Galati, Romania; 7 Clinical Medical Department, Faculty of Medicine and Pharmacy, “Dunarea de Jos” University, 800008 Galati, Romania

**Keywords:** oral–gut–brain axis, periodontitis, neuroinflammation, salivary biomarkers, *Porphyromonas gingivalis*, short-chain fatty acids, blood–brain barrier, microglial activation, dysbiosis, low-grade chronic inflammation

## Abstract

**Background:** Periodontitis affects approximately 7–11% of the global adult population in its severe forms and has been epidemiologically associated with cardiovascular, cardiometabolic, and neurodegenerative diseases. Low-grade chronic inflammation represents the unifying mechanism; however, an integrative framework connecting the oral cavity, the gut, and the brain into a single mechanistic continuum is lacking. **Objective:** This narrative review, conducted with structured (but non-systematic) elements and PRISMA-2020 style reporting used solely as a transparency tool, synthesizes current evidence on the oral–gut–brain axis. A comprehensive literature search was conducted in PubMed/MEDLINE, Scopus, Web of Science, and Google Scholar (2000–March 2026), yielding 159 included studies after structured screening and eligibility assessment. The review focuses on: the molecular mechanisms by which periodontal dysbiosis may disrupt intestinal homeostasis and contribute to neuroinflammation; the role of salivary and intestinal biomarkers as monitoring tools for the entire axis; and emerging pharmacological opportunities targeting this tripartite pathway. **Results:** Periodontal pathogens, particularly *Porphyromonas gingivalis* (*P. gingivalis*) and *Fusobacterium nucleatum*, have been detected ectopically in the gut and are associated with reduced tight junction protein expression and altered Firmicutes/Bacteroidetes ratios in preclinical and observational studies. These perturbations have been associated with increased blood–brain barrier (BBB) permeability, microglial activation, and amyloid-beta (Aβ) accumulation, although causal directionality in humans remains to be established. Salivary biomarkers (MMP-8, IL-1β, IL-6, BDNF) and intestinal biomarkers (short-chain fatty acids, calprotectin) reflect systemic inflammatory burden and offer potential for non-invasive screening. **Conclusions:** The oral–gut–brain axis provides a plausible unifying framework for understanding comorbidity among periodontal, cardiometabolic, and neurodegenerative diseases; however, current evidence is predominantly associative, and mechanistic extrapolation from preclinical models requires validation in longitudinal human studies. Salivary biomarkers may serve as candidate first-line tools for systemic risk assessment, and pharmacological interventions targeting this axis represent promising investigational directions warranting further clinical evaluation.

## 1. Introduction

### 1.1. Epidemiological Context

Periodontitis, defined as a chronic multifactorial inflammatory disease associated with dysbiotic biofilms leading to progressive destruction of the tooth-supporting apparatus, represents one of the most prevalent chronic diseases worldwide [1,2,3]. According to the Global Burden of Disease (GBD) 2021 study, severe periodontitis remains the sixth most prevalent condition globally, affecting approximately 1.1 billion individuals, with an age-standardized prevalence that has shown limited improvement over three decades [2]. The disease burden is disproportionately concentrated in low- and middle-income countries, where access to preventive and therapeutic oral health care remains limited.

The epidemiological significance of periodontitis extends well beyond the oral cavity. A growing body of evidence has established robust associations between periodontal disease and systemic conditions, including cardiovascular diseases, T2DM, obesity, adverse pregnancy outcomes, and, more recently, neurodegenerative disorders such as Alzheimer’s disease (AD) and Parkinson’s disease (PD) [4,5,6,7,8]. Data from the UK Biobank and other large prospective cohorts have demonstrated that individuals with periodontitis exhibit significantly elevated levels of systemic inflammatory markers, including C-reactive protein (CRP), interleukin-6 (IL-6), and tumor necrosis factor-alpha (TNF-α), which are independently associated with increased risk of cardiometabolic and neurological outcomes [5,9].

The socioeconomic burden of periodontitis-associated multimorbidity is substantial. The intersection of oral inflammatory disease with cardiovascular, metabolic, and neurodegenerative conditions amplifies health care costs, reduces quality of life, and increases mortality risk, particularly among aging populations [2,10]. Population aging in both developed and developing countries acts as an amplifying factor, as the prevalence of both periodontitis and neurodegenerative diseases increases exponentially with advancing age [11,12].

### 1.2. Low-Grade Chronic Inflammation

The concept of inflammaging, introduced by Franceschi and colleagues, describes the chronic, sterile, low-grade inflammatory state that accompanies aging and predisposes individuals to a spectrum of age-related diseases [13]. This phenomenon is characterized by persistently elevated levels of pro-inflammatory cytokines (IL-6, TNF-α, interleukin-1β [IL-1β]), acute-phase proteins (CRP, fibrinogen), and activated innate immune cells, in the absence of overt infection [10,13]. Inflammaging represents a convergence point where multiple chronic conditions, including periodontitis, atherosclerosis, insulin resistance, and neurodegeneration, share common pathogenic pathways.

The mechanistic basis of inflammaging involves multiple interconnected processes: cellular senescence with the senescence-associated secretory phenotype (SASP), mitochondrial dysfunction generating excessive reactive oxygen species (ROS), deficient autophagy, persistent activation of the innate immune system through pattern recognition receptors (PRRs), and dysbiosis of mucosal-associated microbiomes [13,14]. Periodontal infection, through chronic stimulation of Toll-like receptors (TLR2, TLR4) by pathogen-associated molecular patterns (PAMPs) such as lipopolysaccharide (LPS) and gingipains, contributes significantly to the systemic inflammatory burden and may accelerate the inflammaging process [5,15,16].

The concept of metainflammation, metabolically induced inflammation, adds another layer of complexity. Excess adipose tissue produces adipokines and pro-inflammatory mediators that interact synergistically with inflammatory signals derived from the periodontium, creating a self-sustaining cycle of chronic inflammation [5,17]. This convergence of oral, metabolic, and immune pathways provides the theoretical foundation for understanding how periodontal disease can influence distant organ systems, including the gastrointestinal tract and the central nervous system (CNS).

### 1.3. From Bidirectional Axes to a Three-Dimensional Model

The conceptual evolution from isolated disease models to integrated multi-organ axes has progressed through several stages. The oral–systemic axis, established through decades of epidemiological and mechanistic research, demonstrated that periodontal pathogens and their inflammatory mediators enter the systemic circulation through the ulcerated epithelium of periodontal pockets, contributing to endothelial dysfunction, atherogenesis, and metabolic disturbances [4,8,18]. Simultaneously, the gut–brain axis emerged as a bidirectional communication highway involving neural (vagal), humoral (cytokine-mediated), and metabolic (microbial metabolite-mediated) pathways linking gut microbiome composition to brain function, behavior, and susceptibility to neurological diseases [19,20,21,22].

More recently, accumulating evidence suggests that oral pathogens may translocate and colonize the gut, thereby modifying the composition and function of the intestinal microbiome, bridging these two paradigms into a proposed oral–gut–brain axis [23,24,25,26,27]. This three-dimensional model hypothesizes that periodontal dysbiosis may not only contribute to systemic inflammation via the hematogenous route but may also act through gastrointestinal colonization, disruption of intestinal barrier integrity, alteration of short-chain fatty acid (SCFA) metabolism, and downstream neuroinflammatory cascades, pathways supported primarily by preclinical and observational data [25,28].

Existing reviews have typically addressed individual segments of this axis in isolation: periodontal disease and systemic inflammation, gut–brain communication in neurodegeneration, or oral microbiome translocation to the gut [24,29,30,31]. The present review addresses this gap by providing a comprehensive, integrative analysis of the entire oral–gut–brain axis, from the initial dysbiotic event in the periodontal pocket to the final neuropathological consequences in the CNS, while simultaneously evaluating the translational potential of biomarkers and pharmacological interventions.

### 1.4. Central Hypothesis and Scope of the Review

The central hypothesis of this review is that periodontal dysbiosis may initiate an inflammatory cascade that, through disruption of the intestinal microbiome and the blood–brain barrier (BBB), contributes to neuroinflammation and neurodegeneration, and that salivary and intestinal biomarkers may serve as non-invasive monitoring tools for this process. Specifically, we propose, on the basis of current preclinical and associative evidence, that: (1) periodontal keystone pathogens, particularly *P. gingivalis* and *F. nucleatum*, ectopically colonize the gut through saliva swallowing, disrupting intestinal microbial ecology and intestinal barrier function; (2) the resulting endotoxemia and altered SCFA metabolism may compromise BBB integrity and shift the microglial phenotype toward a pro-inflammatory state; (3) this neuroinflammatory milieu has been associated in preclinical models with Aβ aggregation, tau hyperphosphorylation, and alpha-synuclein propagation; and (4) biomarkers measurable in saliva and feces may reflect the inflammatory status across all three compartments of the axis [23,25,27,32].

**Figure 1:**
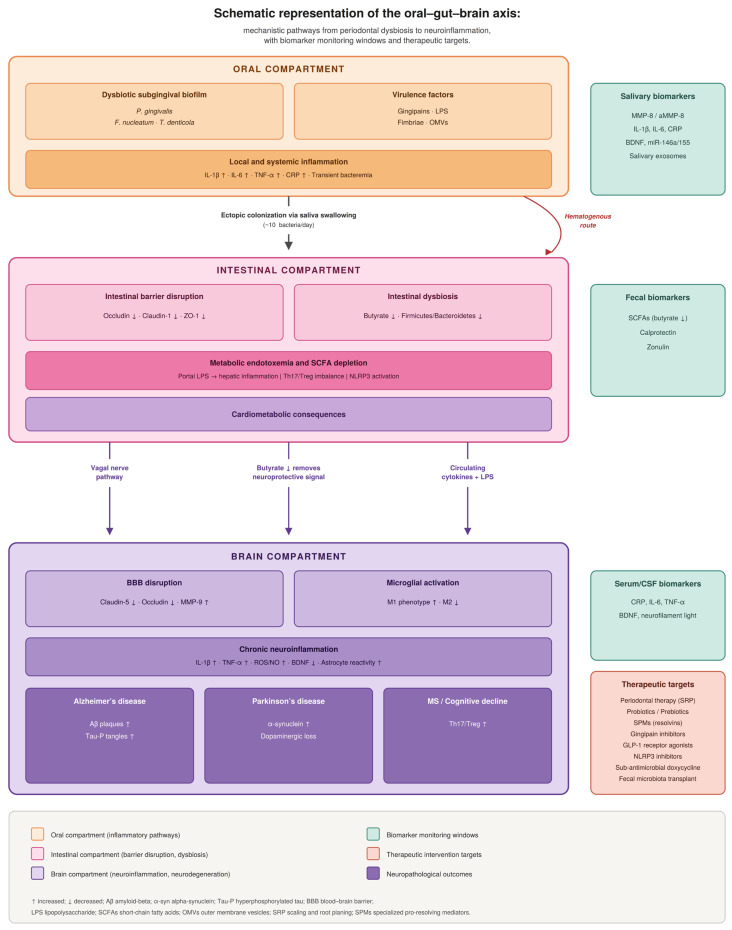
Schematic representation of the oral–gut–brain axis: mechanistic pathways from periodontal dysbiosis to neuroinflammation, with biomarker monitoring windows and therapeutic targets. The oral compartment (upper panel) illustrates the dysbiotic subgingival biofilm dominated by keystone pathogens (*P. gingivalis*, *F. nucleatum*) and their virulence factors, leading to local and systemic inflammation. Ectopic colonization of the gut via saliva swallowing and hematogenous dissemination has been associated with intestinal barrier disruption and dysbiosis (middle panel), resulting in metabolic endotoxemia and short-chain fatty acid (SCFA) depletion in preclinical models. These perturbations are hypothesized to compromise BBB integrity and activate microglia toward a pro-inflammatory phenotype (lower panel), potentially promoting neuropathological cascades in AD, PD, and other neurodegenerative conditions. Biomarker monitoring windows (green panels) indicate non-invasive salivary, fecal, and serum markers reflecting inflammatory status across each compartment. Therapeutic targets highlight pharmacological and microbiome-based interventions at multiple levels of the axis.

The overall mechanistic framework of the oral–gut–brain axis, including the key pathways connecting the three compartments, biomarker monitoring windows, and therapeutic targets addressed in this review, is schematically represented in Figure 1.

## 2. Methodology

**Figure 2:**
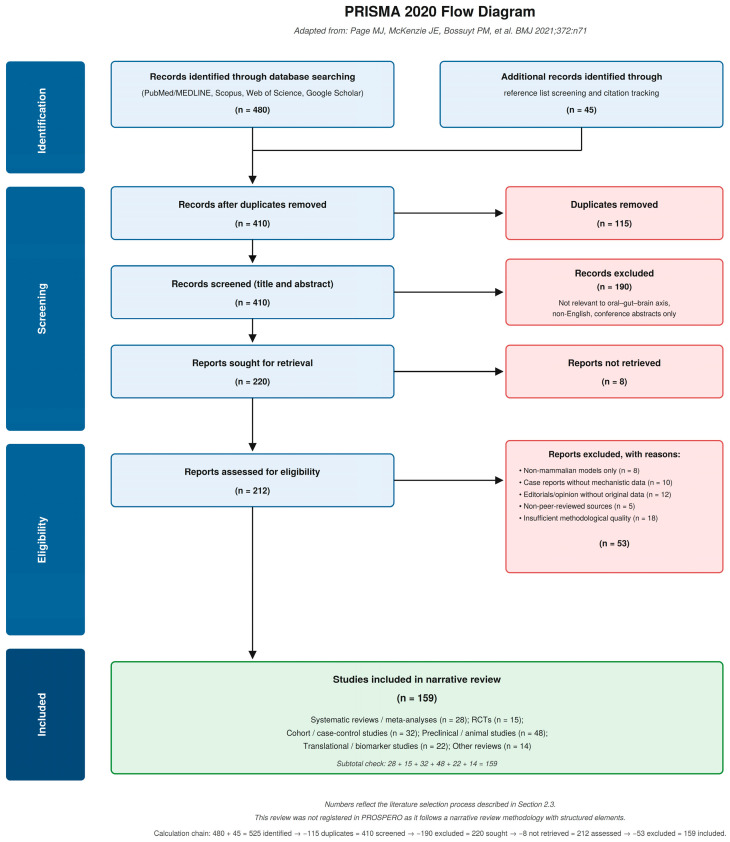
PRISMA 2020 flow diagram of the literature selection process. Of 525 records initially identified through database searching (PubMed/MEDLINE, Scopus, Web of Science, Google Scholar; n = 480) and citation tracking (n = 45), 159 studies were ultimately included in the narrative synthesis. Adapted from Page et al., BMJ 2021;372:n71 [33]. The PRISMA framework was used solely as a transparency-reporting tool and does not imply systematic review methodology.

This narrative review was conducted following a structured but non-systematic approach, with reporting elements adapted from the PRISMA 2020 statement [33] used solely as a transparency tool and not as an indicator of systematic review methodology. The review was not registered in PROSPERO or any equivalent registry, as it does not fulfill the methodological requirements of a full systematic review (no pre-specified protocol, no formal risk-of-bias assessment, and no quantitative synthesis). The study selection process is illustrated in Figure 2 (PRISMA 2020 flow diagram), and a completed PRISMA 2020 checklist is provided as Supplementary Table S1. The PRISMA 2020 statement [33] is cited as a methodological reference and is not included among the 159 studies identified through the literature search. The absence of protocol registration and risk-of-bias assessment represents a recognized limitation of this work and is addressed in the Limitations section. Although PROSPERO registration is not strictly mandatory for narrative reviews, prospective registration would have enhanced methodological transparency, particularly given the hybrid structured approach employed. Future reviews addressing this topic should consider registering a protocol in PROSPERO or an equivalent platform prior to data collection, as this practice is increasingly recommended for all evidence synthesis approaches to improve reproducibility and reduce reporting bias.

### 2.1. Search Strategy

A comprehensive literature search was conducted in multiple electronic databases, including PubMed/MEDLINE, Scopus, Web of Science, and Google Scholar. The search period covered publications from 2000 to March 2026, with particular emphasis on the last five years (2021–2026) to capture the rapidly evolving evidence base. The search strategy used combinations of Medical Subject Headings (MeSH) terms and free-text keywords, including: periodontal disease, periodontitis, oral dysbiosis, oral–gut axis, gut–brain axis, oral–gut–brain axis, neuroinflammation, low-grade inflammation, salivary biomarkers, intestinal biomarkers, *P. gingivalis*, gingipains, *Fusobacterium nucleatum*, short-chain fatty acids, butyrate, BBB, microglial activation, microbiome dysbiosis, AD, PD, calprotectin, MMP-8, and zonulin. Boolean operators (AND, OR) were used for systematic combination of search terms.

### 2.2. Inclusion and Exclusion Criteria

Studies were included if they met the following criteria: systematic reviews, meta-analyses, randomized clinical trials, prospective and retrospective cohort studies, case–control studies, experimental studies on validated animal models, and translational biomarker studies investigating any component of the oral–gut–brain axis. Articles published in English and available as full text were eligible. Exclusion criteria comprised: isolated case reports without a mechanistic perspective, studies conducted exclusively on non-mammalian models, articles published in non-peer-reviewed sources, conference abstracts without accompanying complete manuscripts, and editorials or opinion articles without original data or systematic methodology.

### 2.3. Literature Selection and Synthesis

The literature selection process followed a structured approach: initial screening of titles and abstracts, followed by full-text evaluation of potentially eligible articles and final inclusion based on relevance and quality assessment. As this is a narrative review, no formal meta-analytic framework or risk-of-bias assessment was applied; evidence from different study designs (animal models, observational studies, randomized trials, and systematic reviews) is synthesized narratively and, where relevant, differentiated by level of evidence within the text. Article screening was performed primarily by the lead author; discrepancies in eligibility were resolved by discussion among co-authors. This single-reviewer screening represents a methodological limitation inherent to the narrative review design. However, systematic reviews and meta-analyses identified during the search were prioritized as sources of high-level evidence. Reference lists of included articles were manually searched for additional relevant publications. A total of 525 records were initially identified (480 from database searching and 45 from citation tracking), of which 159 studies were included in the final narrative synthesis after structured screening and eligibility assessment. The complete study selection process is presented in Figure 2 (PRISMA 2020 flow diagram). The synthesis integrates evidence from multiple domains, microbiology, immunology, neuroscience, gastroenterology, and clinical dentistry, to construct a coherent mechanistic narrative. Throughout the synthesis, a clear distinction is maintained between mechanistic insights derived from animal models and in vitro experiments, which permit causal inference within their experimental context, and findings from human observational and cross-sectional studies, which can establish association but not causality. Readers should interpret data from gnotobiotic murine models, ligature-induced periodontitis experiments, and in vitro cell culture studies as providing biological plausibility for the proposed mechanisms, while human epidemiological and clinical data are presented as associative evidence requiring confirmation in prospective interventional designs.

## 3. Oral Dysbiosis and Periodontal Inflammation

In summary, this section describes the transition from a balanced oral microbiome (eubiosis) to a dysbiotic, pro-inflammatory community dominated by keystone pathogens (notably *P. gingivalis*), the principal virulence factors involved (gingipains, lipopolysaccharide, fimbriae, and capsule), and the local-to-systemic inflammatory cascade that ensues.

### 3.1. The Oral Microbiome: From Eubiosis to Dysbiosis

The human oral cavity harbors one of the most complex and diverse microbial communities in the body, comprising approximately 700 cultivable species and an even greater number identified through culture-independent molecular techniques [34,35]. Under healthy conditions, the oral microbiome exists in a state of eubiosis, characterized by a balanced community dominated by commensal species from the genera *Streptococcus*, *Actinomyces*, *Veillonella*, and *Neisseria*, which maintain homeostatic interactions with the host immune system [34,36]. This symbiotic equilibrium involves complex inter-species interactions, including metabolic cooperation, quorum sensing, and competitive exclusion of potential pathogens [28,37].

The transition from eubiosis to dysbiosis is a gradual process driven by both endogenous and exogenous risk factors. Modifiable risk factors include smoking, poorly controlled diabetes mellitus, obesity, psychological stress, inadequate oral hygiene practices, and dietary factors [3,38,39]. Non-modifiable risk factors include genetic susceptibility, advancing age, and sex-related differences in immune response [3,40]. The dysbiotic shift is characterized by a proportional increase in Gram-negative, anaerobic, and proteolytic species at the expense of saccharolytic commensals, creating a microbial community with enhanced pathogenic potential [7,41].

The keystone pathogen hypothesis, proposed by Hajishengallis and colleagues, provides a mechanistic framework for understanding how dysbiosis develops even in the presence of a numerically dominant commensal community [42]. According to this model, *P. gingivalis*, although present in relatively low abundance, subverts host immune defenses through specific virulence mechanisms, thereby remodeling the commensal community into a dysbiotic one with enhanced inflammatory capacity [1,7,42]. This paradigm shift moved the field from the classical specific plaque hypothesis toward a more nuanced understanding of polymicrobial synergy and community-level pathogenesis [37,43].

### 3.2. Virulence Factors of P. gingivalis

#### 3.2.1. Gingipains (Kgp, RgpA, RgpB)

Gingipains are cysteine proteases produced by *P. gingivalis* that represent the bacterium’s most potent virulence arsenal. Two classes exist: arginine-specific gingipains (RgpA and RgpB) and lysine-specific gingipain (Kgp) [44]. These enzymes degrade a broad spectrum of host proteins, including complement components (C3, C5), immunoglobulins (IgA, IgG), cytokines (IL-8, TNF-α), antimicrobial peptides, and extracellular matrix proteins (collagen, fibronectin, laminin) [6,42]. By subverting complement, gingipains prevent efficient opsonization and phagocytosis, allowing *P. gingivalis* to evade innate immune clearance [1,7].

The clinical significance of gingipains extends far beyond the oral cavity. Dominy et al. (2019) detected gingipains in the post-mortem brain tissue of AD patients, with gingipain load correlating with tau pathology and ubiquitin burden [32]. While this cross-sectional post-mortem study cannot establish causation, it provided an important molecular association between periodontal infection and neuropathological features and informed the development of small-molecule gingipain inhibitors as investigational therapeutic agents for AD [32]. Subsequent experimental studies suggested that gingipains may degrade BBB tight junction proteins, facilitate bacterial invasion of endothelial cells, and activate neuroinflammatory cascades in microglial cells, though these findings derive primarily from in vitro and animal models [45,46].

#### 3.2.2. Lipopolysaccharide (LPS)

*P. gingivalis* LPS exhibits structural heterogeneity that confers unique immunomodulatory properties, distinct from those of classical Gram-negative LPS (e.g., *E. coli* LPS). While conventional LPS primarily activates TLR4/MD-2 signaling, *P. gingivalis* LPS can engage both TLR4 and TLR2, with the relative activation depending on the lipid A structure, which can be tetra- or penta-acylated [6]. This dual receptor engagement allows *P. gingivalis* to modulate rather than maximally stimulate the innate immune response, promoting a chronic, low-grade inflammatory state rather than an acute, self-resolving one [1,7].

LPS-mediated activation of the TLR4/MyD88/NF-κB signaling cascade induces the production of pro-inflammatory cytokines (IL-1β, IL-6, TNF-α), chemokines (CXCL8/IL-8, CCL2/MCP-1), and prostaglandins (PGE2) by macrophages, dendritic cells, and epithelial cells [6,7]. Chronic exposure to low concentrations of periodontal LPS entering the systemic circulation through the ulcerated pocket epithelium (~8–20 cm^2^ of ulcerated surface in moderate-to-severe periodontitis) constitutes a persistent source of endotoxemia that amplifies hepatic CRP production, activates endothelial cells, and contributes to the metainflammatory state [1,15].

#### 3.2.3. Fimbriae and Capsule

*P. gingivalis* expresses major (FimA) and minor (Mfa1) fimbriae that mediate adhesion to host epithelial cells, co-aggregation with other bacterial species, and biofilm formation [7,28]. FimA fimbriae interact with multiple host receptors, including integrins (α1β5), complement receptor 3 (CR3/CD11b-CD18), and CXCR4, allowing the bacterium to invade epithelial cells, endothelial cells, and macrophages [1,7]. Intracellular survival in macrophages (the “Trojan horse” mechanism) enables *P. gingivalis* to disseminate systemically while evading extracellular immune defenses [1]. The capsule provides additional resistance against phagocytosis and complement-mediated killing, contributing to bacterial persistence in periodontal lesions and potentially at distant sites [7].

### 3.3. Local Inflammatory Response and Transition to Systemic Inflammation

The local inflammatory cascade in periodontitis follows a well-characterized sequence: initial neutrophil infiltration in response to biofilm challenge, followed by macrophage and dendritic cell activation, T helper cell polarization (predominantly Th1/Th17), B cell activation with antibody production, and ultimately osteoclast-mediated alveolar bone resorption [1,3,7]. The tissue destruction characteristic of periodontitis results from an imbalance between microbial challenge and the host immune response, where excessive or dysregulated inflammation causes collateral damage to periodontal tissues [3,38].

Transient bacteremia is a well-documented consequence of periodontal disease. Daily activities such as toothbrushing, mastication, and professional dental procedures in patients with periodontitis lead to translocation of oral bacteria into the bloodstream through the ulcerated sulcular epithelium [8,47]. Although most episodes of bacteremia are rapidly cleared by the reticuloendothelial system, repeated and chronic exposure to oral pathogens and their products maintains a persistent state of low-grade systemic inflammation [4,5]. This systemic inflammatory amplification is evidenced by elevated circulating levels of CRP, fibrinogen, and multiple pro-inflammatory cytokines in periodontitis patients compared with periodontally healthy controls [4,48,49]. Furthermore, the balance between ROS generated during the inflammatory response and endogenous antioxidant defenses in the oral cavity plays a critical role in modulating the severity of periodontal tissue destruction and its systemic consequences; both endogenous and exogenous sources of ROS contribute to this redox imbalance, and plant-derived antioxidants have been proposed as adjunctive modulators of the oral inflammatory milieu [50].

## 4. The Oral–Gut Axis: Intestinal Colonization and Homeostasis Disruption

In summary, this section outlines how oral pathogens may reach the gut (predominantly via saliva swallowing), compromise intestinal barrier integrity, and induce dysbiosis with downstream cardiometabolic consequences. The supporting evidence is largely derived from preclinical (gnotobiotic and ligature-induced) models and from observational human studies; direct causal demonstration of oral-to-gut translocation in humans is still lacking.

### 4.1. Mechanisms of Oral-to-Gut Translocation

The primary route of oral bacterial translocation to the gastrointestinal tract is saliva swallowing, which occurs approximately 600 times per day, delivering an estimated 1.5 L of saliva containing approximately 10^9^ bacteria daily to the stomach and intestines [51,52,53]. Under normal physiological conditions, the gastric acid barrier (pH 1.5–3.5) eliminates most ingested microorganisms. However, several periodontal pathogens, including *P. gingivalis* and *F. nucleatum*, have demonstrated the ability to survive gastric transit and establish viable colonies in the lower gastrointestinal tract [54,55,56].

Gnotobiotic murine models have provided preclinical evidence suggesting intestinal colonization by oral pathogens. Arimatsu et al. (2014) reported that oral administration of *P. gingivalis* to mice induced significant alterations in gut microbiota composition, increased intestinal inflammation, and promoted metabolic endotoxemia [52]. Nakajima et al. (2015) further showed that orally administered *P. gingivalis* colonized the gut, disrupted barrier function, and facilitated dissemination of enterobacteria to the liver [53]. These findings were corroborated by Kato et al. (2018), who reported that oral administration of *P. gingivalis* altered both the gut microbiome and serum metabolome [55].

Observational evidence consistent with oral-to-gut microbial translocation comes from 16S rRNA sequencing studies demonstrating enrichment of oral cavity-associated taxa in the fecal microbiome of patients with periodontitis; however, these associations do not establish active translocation or causality [51,52,56]. Schmidt et al. (2019) used strain-level metagenomic analysis to show patterns of microbial transmission along the gastrointestinal tract [51]. Lu et al. (2022) reported that periodontitis-related salivary microbiota was associated with exacerbated AD pathology through gut–brain axis crosstalk in animal models, though translation to human disease remains speculative [27].

### 4.2. Disruption of the Intestinal Barrier (“Leaky Gut”)

The intestinal epithelial barrier is maintained by a complex of tight junction (TJ) proteins, including occludin, claudins (particularly claudin-1, -3, -4, -5, and -7), zonula occludens proteins (ZO-1, ZO-2, ZO-3), and junctional adhesion molecules (JAMs), which together regulate paracellular permeability [9,57,58]. Disruption of this barrier, commonly termed “leaky gut,” allows translocation of luminal contents, including bacterial products (LPS, peptidoglycan), intact bacteria, and dietary antigens, into the lamina propria and systemic circulation [57,59].

Periodontal pathogens compromise intestinal barrier integrity through multiple mechanisms. *P. gingivalis* gingipains have been shown in experimental models to cleave tight junction proteins through their proteolytic activity, reducing occludin and claudin-1 expression at the protein level [55,56,60]. Xi et al. (2024) reported that oral administration of *P. gingivalis* exacerbated intestinal barrier dysfunction through activation of NLRP3/IL-1β signaling in intestinal epithelial cells [60]. Additionally, *P. gingivalis* LPS has been shown in preclinical models to activate NF-κB and MAPK signaling pathways in enterocytes, reducing TJ protein expression and increasing paracellular permeability [55,60]. The associated increase in intestinal permeability may facilitate LPS translocation into portal and systemic circulation, contributing to a state of metabolic endotoxemia [15,54].

### 4.3. Oral Pathogen-Induced Intestinal Dysbiosis

Ectopic colonization of the gut by oral pathogens induces profound alterations in gut microbiome composition and function. Studies in both animal models and human cohorts have consistently demonstrated alterations of the Firmicutes-to-Bacteroidetes ratio, with a relative decrease in butyrate-producing species (*Faecalibacterium prausnitzii*, *Roseburia* spp., *Eubacterium rectale*) and an increase in pro-inflammatory Gram-negative species [22,54,56,61]. The competitive displacement and metabolic interference caused by intruding oral pathogens disrupt the ecological balance of the commensal intestinal microbiota, leading to a self-propagating dysbiotic cycle [51,52,62].

The functional consequences of oral pathogen-induced intestinal dysbiosis may be particularly evident in the altered metabolism of short-chain fatty acids (SCFAs). Butyrate, the primary energy source for colonocytes and a potent anti-inflammatory mediator, is reduced in dysbiotic states in preclinical and observational studies, while acetate and propionate ratios shift unfavorably [63,64,65]. This SCFA imbalance has been associated with impaired colonocyte function, weakened mucosal barrier integrity, and reduced regulatory T cell (Treg) differentiation, while simultaneously promoting Th17 polarization and exacerbating the Th17/Treg imbalance that characterizes chronic mucosal inflammation, although the extent to which oral pathogen colonization specifically drives these changes in humans remains unclear [21,62,63,66].

### 4.4. Cardiometabolic Consequences

Intestinal barrier dysfunction and the resulting endotoxemia from oral pathogen-induced intestinal disruption have significant cardiometabolic implications. Portal venous delivery of bacterial LPS to the liver activates hepatic Kupffer cells (resident macrophages) through TLR4 signaling, promoting hepatic inflammation, steatosis, and insulin resistance [15,55,67]. Lyu et al. (2025) demonstrated that periodontal infection with *P. gingivalis* was associated with aggravated nonalcoholic steatohepatitis through intestinal barrier disruption and hepatic inflammatory activation in animal models [67]. This metabolic endotoxemia has been proposed as a contributor to the insulin resistance and dyslipidemia observed in periodontitis patients, representing a plausible mechanistic link in the bidirectional relationship between periodontal disease and type 2 diabetes [48,68,69].

Endothelial activation by circulating LPS and pro-inflammatory cytokines has been associated with atherogenic processes, including upregulation of adhesion molecules (ICAM-1, VCAM-1, E-selectin), monocyte recruitment, foam cell formation, and plaque instability [4,8,70]. A bidirectional relationship between intestinal dysbiosis, obesity, and metainflammation has been proposed, whereby each component may exacerbate the others, including periodontal disease severity, through altered immune function and metabolic disruption, suggesting a self-sustaining but not yet causally proven inflammatory cascade [5,15,71].

## 5. The Gut–Brain Axis: From Intestinal Disruption to Neuroinflammation

In brief, gut–brain communication operates through three complementary routes, neural (vagal), humoral (cytokine- and endotoxin-mediated), and microbial-metabolite-driven, any of which may be perturbed by intestinal dysbiosis arising upstream from oral pathogen translocation. The following subsections outline these pathways, the consequences for blood–brain barrier integrity, and the resulting microglial response. The mechanistic links described below derive predominantly from preclinical and observational evidence, and should not be interpreted as causally established in humans.

### 5.1. Communication Pathways of the Gut–Brain Axis

#### 5.1.1. The Neural Pathway: The Vagus Nerve

The vagus nerve (cranial nerve X) constitutes the primary neural conduit between the gastrointestinal tract and the CNS. Vagal afferent neurons, whose cell bodies reside in the nodose ganglion, terminate in the nucleus tractus solitarius (NTS) of the brainstem and are capable of sensing luminal cytokines, bacterial metabolites, and neuroactive compounds produced by the intestinal microbiota [31,72,73]. From the NTS, signals are transmitted to higher brain centers, including the hypothalamus, amygdala, and prefrontal cortex, through ascending projections that modulate emotion, cognition, and neuroendocrine function [74].

The vagal pathway has been demonstrated as essential for transmitting gut-derived pathological signals to the brain. Studies showing that vagotomy prevents the behavioral and neurochemical effects of probiotic administration [72] and attenuates the propagation of alpha-synuclein pathology from the gut to the brain in PD models [75,76] underscore the importance of this pathway. In the context of the oral–gut–brain axis, intestinal inflammation induced by ectopic colonization of oral pathogens can activate vagal afferents through local cytokine release, thereby communicating the inflammatory state to the CNS [31].

#### 5.1.2. The Humoral/Circulatory Pathway

Pro-inflammatory cytokines generated in the intestinal mucosa (IL-6, TNF-α, IL-1β) can enter the systemic circulation and reach the brain through several mechanisms: active transport across the BBB via specific cytokine transporters, passage through circumventricular organs (CVOs) lacking a complete BBB (area postrema, median eminence, subfornical organ), and direct BBB disruption through endothelial cell activation [77,78,79]. Systemic LPS, derived from the increased intestinal permeability described above, activates cerebral endothelial TLR4, inducing local production of inflammatory mediators and recruitment of peripheral immune cells into the CNS [77,80].

The humoral pathway is particularly relevant for the oral–gut–brain axis because the combination of periodontal bacteremia and intestinal endotoxemia may create a dual source of systemic inflammatory signaling. It has been hypothesized that circulating cytokines produced by both activated periodontal tissues and inflamed intestinal mucosa converge on the BBB and brain parenchyma, potentially amplifying the neuroinflammatory response beyond what either source would produce individually, though this additive effect has not been directly quantified in humans [38,81].

#### 5.1.3. The Microbial Metabolite Pathway

Microbial metabolites, particularly SCFAs, tryptophan derivatives, and bile acid metabolites, represent a third major communication pathway between the gut and the brain [63,64,65,82]. Butyrate, the most extensively studied SCFA in the context of neuroprotection, crosses the BBB via monocarboxylate transporters (MCTs) on endothelial cells and exerts anti-inflammatory effects through histone deacetylase (HDAC) inhibition and activation of free fatty acid receptors (FFAR2/GPR43 and FFAR3/GPR41) [63,64,83]. Within the CNS, butyrate suppresses microglial activation, promotes expression of neurotrophic factors (including brain-derived neurotrophic factor [BDNF]), and maintains BBB integrity by upregulating tight junction protein expression [63,83,84].

The reduction of butyrate-producing bacteria consequent to oral pathogen-induced intestinal dysbiosis therefore removes a critical neuroprotective signal. Additionally, tryptophan metabolism by intestinal bacteria generates bioactive compounds through the indole and kynurenine pathways. The aryl hydrocarbon receptor (AhR), activated by microbial indole derivatives, modulates astrocyte and microglial function, while the balance between neuroprotective (kynurenic acid) and neurotoxic (quinolinic acid) metabolites of the kynurenine pathway is disrupted in dysbiosis, potentially contributing to excitotoxicity and neurodegeneration [82,85,86].

### 5.2. Blood–Brain Barrier (BBB) Disruption

The BBB is a highly specialized neurovascular structure composed of brain microvascular endothelial cells (BMECs), pericytes, astrocytic end-feet, and a basement membrane, forming the neurovascular unit (NVU) [78,79]. BBB integrity depends on the proper expression and assembly of tight junction proteins, particularly claudin-5, occludin, and ZO-1, which restrict paracellular permeability to ions and macromolecules [78,79]. Multiple mechanisms identified in the oral–gut–brain axis converge on BBB disruption.

First, matrix metalloproteinases (MMP-2, MMP-9), released by activated microglia and infiltrating leukocytes, degrade tight junction proteins and basement membrane components [79,87]. Second, *P. gingivalis* gingipains have demonstrated the ability to directly cleave BBB tight junction proteins in experimental models [32,45]. Third, systemic LPS activates endothelial TLR4, inducing oxidative stress through NADPH oxidase activation and impairing tight junction assembly [77,80]. Fourth, the reduction in butyrate consequent to intestinal dysbiosis removes the tonic protective signal that normally upregulates claudin-5 and occludin expression in cerebral endothelium [83,88]. Outer membrane vesicles (OMVs) of *P. gingivalis*, smaller than intact bacteria, can traverse the BBB through degradation of endothelial adhesion proteins, including PECAM-1 and β1-integrin [35]. Collectively, these mechanisms increase BBB permeability to circulating inflammatory mediators, bacteria, and bacterial products, initiating or exacerbating neuroinflammation [78,79].

### 5.3. Neuroinflammation: Microglia as the Central Pivot

Microglia, the resident innate immune cells of the CNS, serve as primary sentinels and effectors of neuroinflammatory responses [89,90,91]. Under homeostatic conditions, microglia maintain a ramified, surveying morphology, continuously monitoring the brain parenchyma for pathological signals through a repertoire of pattern recognition receptors (TLRs, NOD-like receptors, scavenger receptors) [89,91]. Upon activation by inflammatory stimuli, microglia undergo phenotypic transformation: the classical M1 phenotype produces pro-inflammatory cytokines (IL-1β, IL-6, TNF-α), reactive oxygen and nitrogen species (ROS, NO), and chemokines, while the alternative M2 phenotype promotes tissue repair, anti-inflammatory cytokine production (IL-10, TGF-β), and phagocytic clearance of debris [87,90,92,93].

In the context of the oral–gut–brain axis, multiple convergent signals have been proposed to drive microglial polarization toward the pro-inflammatory M1 phenotype: circulating LPS engaging microglial TLR4 [80]; pro-inflammatory cytokines traversing a disrupted BBB [77,81]; gingipains and other bacterial virulence factors potentially reaching the brain parenchyma [32,45]; and loss of butyrate-mediated HDAC inhibition that normally maintains microglial homeostasis [63,83,94]. Experimental evidence suggests that chronic microglial activation may sustain a neuroinflammatory milieu through autocrine and paracrine amplification loops involving microglia–astrocyte crosstalk, where reactive astrocytes may further potentiate cytokine release and impair synaptic function, though these mechanisms have been established primarily in animal models and cell culture [87,90,93].

### 5.4. Neuropathological Consequences

#### 5.4.1. Alzheimer’s Disease

AD, the most prevalent form of dementia affecting approximately 55 million people worldwide, is characterized by extracellular amyloid-beta (Aβ) plaques, intracellular neurofibrillary tangles composed of hyperphosphorylated tau protein (Tau-P), synaptic loss, and progressive neuronal death [19,95,96]. Neuroinflammation driven by oral–gut–brain axis disruption promotes AD pathology through several interconnected mechanisms: (1) activation of β- and γ-secretases by pro-inflammatory cytokines intensifies amyloidogenic processing of amyloid precursor protein (APP), increasing Aβ production [93,95,97]; (2) microglial activation impairs phagocytic clearance of Aβ while simultaneously promoting the release of neurotoxic inflammatory mediators [90,93,96]; (3) tau hyperphosphorylation is stimulated by GSK-3β activation downstream of inflammatory signaling cascades [95,98].

Evidence associating periodontal pathogens with AD neuropathology includes the detection of *P. gingivalis* DNA and gingipains in the post-mortem brain tissue of AD patients, with gingipain load correlating with tau and ubiquitin pathology [32,99]. Studies in AD transgenic mice have reported that ligature-induced periodontitis or oral administration of *P. gingivalis* was associated with exacerbated Aβ deposition, tau pathology, and cognitive decline in rodent models [100,101,102,103]. Kanagasingam et al. (2022) showed that *P. gingivalis*-conditioned medium was associated with amyloidogenic processing of APP in neuronal cell lines, providing in vitro mechanistic data [45]. Furthermore, gut microbiota alterations reported in AD patients, including reductions in butyrate producers and increases in LPS-producing species, partially overlap with the dysbiotic patterns observed following oral pathogen colonization in animal models, though a causal relationship has not been established in humans [8,104,105,106,107,108].

#### 5.4.2. Parkinson’s Disease

PD is characterized by the loss of dopaminergic neurons in the substantia nigra pars compacta and the accumulation of alpha-synuclein (α-syn) aggregates (Lewy bodies) in both the central and peripheral nervous systems [109,110]. The Braak hypothesis proposes that PD pathology may initiate in the enteric nervous system (ENS) and propagate retrogradely through the vagus nerve to the brainstem and subsequently to higher brain regions [110]. This staging framework aligns closely with the oral–gut–brain axis model, as intestinal dysbiosis induced by oral pathogens could represent an upstream trigger for enteric α-syn aggregation [75,76,109].

Preclinical evidence reviewed by Socała et al. (2021) supports the requirement of gut microbiota for the development of motor deficits and neuroinflammation in murine PD models, with microbial metabolites promoting α-syn aggregation and microglial activation [75]. Kim et al. (2019) provided direct evidence of transneuronal propagation of pathological α-syn from the gut to the brain via the vagus nerve [76]. Challis et al. (2020) refined this model by demonstrating that α-syn fibrils seeded in the gut promote brain pathology specifically in aged mice, highlighting the intersection of aging, intestinal dysbiosis, and neurodegeneration [111].

#### 5.4.3. Other Neurodegenerative Conditions

Multiple sclerosis (MS), an autoimmune demyelinating disorder, has been linked to alterations in gut microbiota that skew T cell differentiation toward Th17 and away from Treg phenotypes [110,111,112]. The Th17/Treg imbalance induced by oral pathogen-driven intestinal dysbiosis may therefore contribute to the autoimmune milieu driving demyelination [62,110]. Additionally, age-associated cognitive decline, even in the absence of manifest neurodegenerative disease, is accelerated by chronic neuroinflammation (inflammaging), and the oral–gut–brain axis provides a continuous source of inflammatory stimulation that may hasten this process [13,26,81,113].

## 6. Salivary and Intestinal Biomarkers as Axis Monitoring Tools

In summary, this section reviews salivary and fecal markers along a gradient of validation: clinically validated markers (e.g., salivary MMP-8/aMMP-8, fecal calprotectin), which have established utility for periodontal or intestinal inflammation; and emerging or investigational markers (e.g., salivary BDNF, fecal SCFA profiling, miRNA signatures), whose role as predictors of neurodegenerative risk along the oral–gut–brain axis remains exploratory and is not yet supported by adequately powered prospective clinical studies.

### 6.1. Rationale for Non-Invasive Biomarkers

Clinical translation of the oral–gut–brain axis concept requires the identification of accessible, non-invasive biomarkers capable of reflecting the inflammatory status across all three compartments. Saliva, often described as a “mirror of the body,” contains a rich repertoire of proteins, metabolites, nucleic acids, and microbial components that reflect both local oral and systemic inflammatory status [114,115,116]. Advantages of salivary diagnostics include non-invasive collection, the possibility of repeated sampling, patient comfort, reduced infection risk compared with blood collection, and the feasibility of point-of-care (POC) testing in dental and primary care offices [114,117]. Intestinal biomarkers, measured in fecal samples, provide direct information about gut microbiome composition, metabolic function, and mucosal inflammatory status, complementing information obtained from saliva [59].

### 6.2. Validated Salivary Biomarkers

It should be noted that “validated” in this section refers primarily to validation in the context of periodontal disease assessment. Their utility as monitoring tools for the broader oral–gut–brain axis, and, in particular, as predictors of neurodegenerative risk, remains largely investigational and has not been established in adequately powered prospective studies.

#### 6.2.1. Pro-Inflammatory Cytokines (IL-1β, IL-6, TNF-α)

Salivary concentrations of pro-inflammatory cytokines, particularly IL-1β and IL-6, have been extensively validated as biomarkers of periodontal disease severity. Meta-analyses have confirmed that salivary IL-1β and IL-6 levels are significantly elevated in periodontitis patients compared with healthy controls and correlate with clinical parameters including probing depth, clinical attachment loss, and bleeding on probing [114,118]. Beyond their role as periodontal biomarkers, salivary cytokines reflect systemic inflammatory burden and have been associated with cardiovascular risk markers in serum [4,114]. However, diagnostic specificity is limited by the fact that multiple systemic inflammatory conditions can elevate salivary cytokine levels independently of periodontal status [114,115].

#### 6.2.2. Matrix Metalloproteinase-8 (MMP-8)

MMP-8 (collagenase-2) is the most extensively studied and clinically validated salivary biomarker for periodontitis. Produced primarily by neutrophils during the inflammatory response, MMP-8 degrades type I collagen, the principal structural protein of the periodontal ligament, making it a direct marker of ongoing tissue destruction [117,119,120,121]. Recent meta-analyses have confirmed that salivary MMP-8 levels are significantly higher in periodontitis patients, with a standardized mean difference of approximately 2.71 compared with healthy controls [120]. The activated form (aMMP-8) has been proposed as a more specific indicator of active disease, as it reflects ongoing collagenolysis [117,121,122].

Point-of-care testing for aMMP-8 has been commercialized (e.g., the PerioSafe^®^ test, Dentognostics GmbH, Solingen, Germany)), enabling chair-side assessment of periodontal disease activity within minutes [117,122]. Importantly, elevated aMMP-8 has been associated with metabolic syndrome parameters and systemic inflammatory markers, suggesting its potential as a screening tool for systemic inflammatory burden beyond the oral cavity [117,122,123]. This dual local-systemic informational content makes MMP-8/aMMP-8 an attractive candidate for integration into multicompartmental biomarker panels for the oral–gut–brain axis.

#### 6.2.3. Salivary CRP (CRP)

CRP, an acute-phase protein synthesized primarily by hepatocytes in response to IL-6, enters saliva through gingival crevicular fluid transudation and, to a lesser extent, through salivary gland secretion [114]. Salivary CRP levels have been shown to correlate with serum CRP and cardiovascular risk assessment, offering the potential for non-invasive cardiovascular risk screening in dental offices [114]. Lab-on-a-chip platforms and lateral flow immunoassays for salivary CRP quantification are under development [116].

#### 6.2.4. Brain-Derived Neurotrophic Factor (BDNF)

BDNF, a neurotrophin essential for neuronal survival, synaptic plasticity, and cognitive function, is also detectable in saliva. Haririan et al. (2022) reported reduced salivary BDNF levels in periodontitis patients in a single study, suggesting a possible link between oral inflammation and neurotrophic factor depletion; however, this finding has not been independently replicated and must be interpreted with caution [124]. BDNF represents a conceptual bridge between the oral and cerebral components of the axis, as both periodontal inflammation and intestinal dysbiosis-induced butyrate reductions have been associated with hippocampal BDNF expression in animal models [63,124]. Salivary BDNF should currently be considered an exploratory, experimental biomarker: clinical validation in adequately powered, prospective cohorts is lacking, and its specificity for neurodegeneration risk has not been established.

#### 6.2.5. Emerging Biomarkers: miRNA, Exosomes, Metabolites

MicroRNAs (miRNAs), small non-coding RNA molecules that regulate post-transcriptional gene expression, have been identified in saliva and show disease-specific signatures. In particular, miR-146a and miR-155, which modulate NF-κB signaling and inflammatory gene expression, are differentially expressed in the saliva of periodontitis patients and represent potential biomarkers of inflammatory regulation [115,125]. Salivary exosomes, membrane-bound extracellular vesicles carrying proteins, lipids, and nucleic acids, can serve as vehicles for intercellular and inter-organ communication and represent a frontier in liquid biopsy diagnostics [116,125]. Metabolomic profiling of saliva, including analysis of fatty acids, amino acids, and organic acids, is emerging as a high-throughput approach for capturing the metabolic signature of oral–systemic disease interactions [115,116].

### 6.3. Intestinal (Fecal) Biomarkers

#### 6.3.1. Short-Chain Fatty Acids (SCFAs)

Fecal SCFA profiling, particularly quantification of butyrate, acetate, and propionate, provides direct information about the metabolic output of the gut microbiome. In the context of oral pathogen-induced intestinal dysbiosis, reduced butyrate and altered butyrate/total SCFA ratios have been documented and correlate with intestinal permeability, systemic inflammation, and, indirectly, neuroinflammatory status [63,64,65]. Enzymatic and chromatographic methods (gas chromatography, HPLC) for fecal SCFA quantification are well established [64].

#### 6.3.2. Fecal Calprotectin

Calprotectin, a calcium-binding protein released by activated neutrophils, is an established clinical marker of intestinal inflammation routinely used in the diagnosis and monitoring of inflammatory bowel diseases (IBD). Fecal calprotectin levels reflect the degree of neutrophilic infiltration in the intestinal mucosa and can serve as an indicator of intestinal inflammation induced by ectopic colonization of oral pathogens [58,59].

#### 6.3.3. Zonulin

Zonulin, a protein that modulates intestinal permeability through reversible disassembly of tight junctions, serves as a serological and fecal marker of intestinal barrier integrity (“leaky gut”) [59]. Elevated fecal or serum zonulin levels indicate increased intestinal permeability and have been associated with autoimmune diseases, metabolic syndrome, and neurological conditions [59]. In the context of the oral–gut–brain axis, zonulin could serve as an indicator of the barrier-disrupting effects of oral pathogen colonization in the gut.

### 6.4. Integrated Multi-Biomarker Panel: Translational Vision

The translational potential of the oral–gut–brain axis concept could be enhanced through the development of integrated, multi-biomarker panels that simultaneously monitor all three compartments. It is important to distinguish between markers at different stages of validation. Clinically validated markers include salivary MMP-8/aMMP-8 (with commercially available POC testing) and IL-1β for the oral compartment, and fecal calprotectin for the intestinal compartment. Emerging or experimental markers, requiring further validation before clinical application, include salivary BDNF, fecal SCFA profiling as a systemic inflammatory proxy, and serum or salivary neuroinflammatory markers (Table 1) [59,63,114,117,124]. We propose, as a research vision rather than a clinical recommendation, a panel architecture combining validated periodontal biomarkers with fecal SCFA and calprotectin, integrated with clinical periodontal scores (PPD, CAL, BOP) and cognitive assessments (MMSE, MoCA). Such a panel would require prospective validation in large cohorts before any claim of clinical utility for risk stratification or neurodegeneration screening could be substantiated. The development of multiplexed POC platforms utilizing microfluidics and biosensor technologies represents an aspirational technological goal, with no currently validated instrument capable of simultaneous multi-compartment measurement [116,117,126].

## 7. Pharmacological and Therapeutic Implications

In summary, the therapeutic strategies discussed below span a spectrum from clinically established interventions (e.g., non-surgical periodontal therapy) to investigational approaches whose efficacy along the oral–gut–brain axis, particularly for neurodegenerative endpoints, remains unproven. With the sole exception of periodontal therapy itself, none of the agents discussed in this section is currently approved or recommended for axis-directed prevention or treatment of neurodegenerative disease, and references to mechanistic plausibility or preclinical efficacy should not be construed as evidence of clinical validation.

### 7.1. Periodontal Therapy as a Systemic Intervention

The hypothesis that periodontal treatment can reduce systemic inflammatory burden is supported by multiple clinical studies demonstrating reductions in serum CRP, IL-6, and TNF-α after non-surgical periodontal therapy (scaling and root planing) [4,48,127,128]. D’Aiuto et al. (2018) showed in a randomized clinical trial that intensive periodontal treatment in patients with T2DM significantly improved glycemic control (HbA1c) and reduced systemic inflammatory markers at 12 months [48]. Czesnikiewicz-Guzik et al. (2019) provided causal evidence from a Mendelian randomization study and a randomized trial showing that periodontal therapy reduces blood pressure in hypertensive patients [49].

Critically, no randomized controlled clinical trial has yet demonstrated that periodontal treatment can prevent or slow the progression of neurodegenerative diseases. Although observational studies suggest that individuals receiving regular periodontal care have reduced dementia risk [129], establishing a causal therapeutic link requires longitudinal interventional trials with cognitive and neuroimaging endpoints. Such trials represent an urgent priority for the field.

### 7.2. Pharmacological Modulation of Inflammation

#### 7.2.1. Anti-Inflammatory Agents with Pleiotropic Potential

Statins (HMG-CoA reductase inhibitors), beyond their established lipid-lowering effects, exhibit anti-inflammatory properties through inhibition of isoprenoid synthesis, reduction in NF-κB activation, and suppression of pro-inflammatory cytokine production [4,130]. These pleiotropic effects have been observed in the CNS in preclinical models, where statins reduced microglial activation and Aβ production; however, randomized clinical trial evidence for neuroprotection in AD or PD remains inconclusive [93,130]. GLP-1 receptor agonists (e.g., exenatide, liraglutide, semaglutide), initially developed for type 2 diabetes, have shown neuroprotective effects in animal models by reducing neuroinflammation, oxidative stress, and apoptosis [131,132,133]. Clinical trials evaluating GLP-1 agonists in AD and PD are ongoing and should be considered investigational; definitive efficacy data from Phase III trials are not yet available [132,134].

Specialized pro-resolving mediators (SPMs), including resolvins, protectins, and maresins, represent a paradigmatic class of endogenous lipid mediators that actively promote the resolution of inflammation rather than merely suppressing it [135,136]. In the context of periodontal disease, resolvins have demonstrated efficacy in reducing alveolar bone loss, promoting tissue regeneration, and attenuating systemic inflammatory responses in preclinical models [137]. Their potential to simultaneously resolve inflammation across all three compartments of the axis makes SPMs particularly attractive candidates for oral–gut–brain axis-directed therapy.

#### 7.2.2. Gingipain Inhibitors

COR388 (atuzaginstat), developed by Cortexyme, represented the first small-molecule gingipain inhibitor to advance to Phase II/III clinical trials for AD [32,134]. The therapeutic rationale was based on the detection of gingipains in AD post-mortem brains and preclinical evidence that gingipain inhibition reduces Aβ production, neuroinflammation, and bacterial load in animal models [32]. The clinical development program was terminated due to hepatotoxicity signals and the failure to meet primary efficacy endpoints, and no gingipain inhibitor is currently approved or in active late-phase clinical development for any neurodegenerative indication [134,138]. While the biological rationale for targeting microbial virulence factors as a disease-modifying strategy remains scientifically interesting, the clinical failure of atuzaginstat underscores the substantial translational gap between preclinical promise and clinical efficacy in this field [134,138].

### 7.3. Microbiome Modulation

#### 7.3.1. Probiotics and Prebiotics

Oral probiotics, particularly strains of *Lactobacillus reuteri*, *L. brevis*, *L. rhamnosus*, and *Bifidobacterium* species, have demonstrated efficacy as adjuncts to non-surgical periodontal therapy, reducing probing depth, clinical attachment loss, and salivary pathogen load in meta-analyses of randomized clinical trials [139,140,141]. Mechanistically, oral probiotics compete with periodontal pathogens for adhesion sites, produce antimicrobial substances (bacteriocins, hydrogen peroxide), and modulate local immune responses toward anti-inflammatory profiles [140,141].

Intestinal probiotics targeting butyrate restoration and gut barrier repair complement oral probiotics in a dual-compartment strategy. Prebiotics, including fructo-oligosaccharides (FOS), galacto-oligosaccharides (GOS), and resistant starch, selectively stimulate the growth and metabolic activity of butyrate-producing commensals, thereby enhancing SCFA production and supporting intestinal barrier integrity [64,140,141,142,143]. Synbiotics combining probiotic strains with prebiotic substrates offer the potential for synergistic effects in both oral and intestinal compartments [142]. Beyond classical prebiotics, dietary polyphenols from plant-based functional foods have demonstrated prebiotic-like properties and anti-inflammatory potential along the gastrointestinal tract. Notably, the stability of phenolic compounds from fermented functional beverages under simulated gastrointestinal conditions has been confirmed, supporting their bioavailability for gut microbiome modulation and their potential role as dietary adjuncts in oral–gut axis interventions [144].

#### 7.3.2. Fecal Microbiota Transplantation (FMT)

FMT, established as first-line therapy for recurrent *Clostridioides difficile* infection [145], has shown promising preclinical results in neurodegenerative disease models. Sun et al. (2018) reported that FMT from healthy donors to MPTP-induced PD mice was associated with restored gut microbiota diversity, reduced microglial activation, and attenuated dopaminergic neurodegeneration through modulation of TLR4/TNF-α signaling in an animal model [146]. Hazan (2020) reported cognitive improvement after FMT in a single patient with AD; this observation cannot be generalized and should be considered anecdotal evidence only [147]. FMT for neurological indications must be considered strictly investigational: challenges of donor standardization, microbiota engraftment variability, safety concerns (including serious adverse events reported in immunocompromised recipients), and the absence of controlled clinical trials preclude any current clinical recommendation [148].

### 7.4. Drug Repurposing and Novel Therapeutic Targets

Minocycline, a tetracycline antibiotic, possesses anti-inflammatory and neuroprotective properties independent of its antimicrobial action, including inhibition of microglial activation, reduction in matrix metalloproteinase activity, and attenuation of caspase-mediated apoptosis [149,150]. Sub-antimicrobial dose doxycycline (SDD, 20 mg twice daily) is FDA-approved as an MMP inhibitor for periodontitis (Periostat^®^) and, at these doses, does not exert antibiotic selective pressure, making it an attractive candidate for long-term anti-inflammatory therapy along the axis [119,149].

NLRP3 inflammasome inhibitors represent a promising drug class targeting a convergence point of inflammatory signaling across all three compartments of the axis. The NLRP3 inflammasome is activated by diverse danger signals, including bacterial LPS, crystalline substances (including Aβ fibrils), and metabolic stress signals, leading to caspase-1 activation and maturation and release of IL-1β/IL-18 [60,151,152,153]. MCC950 and related NLRP3-specific inhibitors have shown efficacy in preclinical models of periodontitis, IBD, and AD, suggesting the potential for a single pharmacological intervention to modulate inflammation simultaneously across oral, intestinal, and cerebral compartments [151,154]. Tryptophan–kynurenine axis modulators, including IDO1 inhibitors and kynurenine aminotransferase (KAT) modulators, represent additional novel targets for restoring neurotransmitter balance disrupted by intestinal dysbiosis [18,82,85]. A summary of the principal pharmacological and therapeutic strategies targeting the oral–gut–brain axis is presented in Table 2.

## 8. Perspectives and Future Research Directions

### 8.1. Knowledge Gaps

Despite the substantial body of evidence reviewed herein, several critical knowledge gaps persist. First, the causal directionality of the oral–gut–brain axis has not been definitively established in humans. Most clinical evidence is correlational or cross-sectional, and although preclinical models provide mechanistic plausibility, translation to human pathophysiology requires confirmation through prospective longitudinal cohort studies and interventional trials [1,5,38]. Second, the quantitative contribution of periodontal disease to total systemic inflammatory burden relative to other sources (adipose tissue, intestinal mucosal inflammation, chronic infections) remains poorly defined [1,127]. Indeed, it must be acknowledged that periodontal disease represents only one component of the total systemic inflammatory burden, alongside major contributors such as visceral adipose tissue, which continuously secretes pro-inflammatory adipokines (TNF-α, IL-6, leptin), and chronic low-grade infections at other mucosal sites. While periodontal disease may act as an amplifying trigger within an already inflamed systemic milieu, its relative quantitative contribution compared to these other sources has not been directly measured in human studies and should not be overstated. Third, standardization of collection, processing, and analysis methods for salivary and fecal biomarkers is essential for clinical implementation [114,120]. This is a particularly critical limitation that deserves greater emphasis. Currently, salivary biomarker collection lacks consensus protocols regarding stimulated versus unstimulated saliva, time of collection, pre-analytical processing conditions (centrifugation speed, storage temperature, freeze–thaw cycles), and reference ranges across laboratories. Similarly, fecal biomarker analysis, particularly for SCFA profiling and calprotectin, is hampered by variability in sample homogenization, extraction methods, and analytical platforms (gas chromatography, HPLC, immunoassay). Until unified, validated protocols are established and widely adopted, multi-center comparability of results will remain limited, constituting a major barrier to clinical translation of the oral–gut–brain axis biomarker panel.

### 8.2. Multi-Omics Integration

The integration of multiple omics platforms, metagenomics (oral and intestinal microbiome), proteomics (salivary and serum biomarkers), metabolomics (SCFAs, tryptophan metabolites, bile acids), and transcriptomics (host gene expression in periodontal tissues and peripheral blood), offers the potential to construct a comprehensive, systems-level understanding of the oral–gut–brain axis [116,126]. Machine learning and artificial intelligence algorithms applied to multi-omics datasets can identify predictive biosignatures for disease risk stratification, treatment response prediction, and personalized therapeutic recommendations [126]. Digital twin models and in silico simulations of the axis represent a future frontier for hypothesis testing and treatment optimization [126].

### 8.3. Personalized Medicine and Integrated Prevention

Translating the oral–gut–brain axis concept into clinical practice requires the integration of periodontal screening into cardiometabolic and neurodegenerative risk assessment frameworks. Salivary biomarker panels, tailored to individual patient profiles (age, comorbidities, microbiome composition, genetic risk), could enable personalized risk stratification and targeted preventive interventions [117,126,153]. The interprofessional collaboration model, connecting dental practitioners, cardiologists, neurologists, gastroenterologists, and pharmacists, is essential for implementing an integrated care approach that addresses the systemic implications of periodontal disease [1,38].

### 8.4. Emerging Technologies

Wearable biosensors capable of continuous or semi-continuous monitoring of salivary biomarkers (pH, cortisol, inflammatory cytokines) are under development and could revolutionize disease monitoring by providing real-time data on inflammatory status [154]. Next-generation POC devices integrating microfluidics, electrochemical detection, and smartphone connectivity offer the potential for home monitoring of oral health linked to clinical decision support systems [116,117,126]. Nanomedicine approaches, including nanoparticle-based drug delivery systems for targeted anti-inflammatory therapy at the intestinal barrier or BBB, and theranostic nanoparticles combining diagnostic and therapeutic functions, represent a technological frontier with the potential to transform the management of axis-related diseases [159,160].

## 9. Limitations

Several important limitations of the current evidence base and of this review itself must be acknowledged. First, the majority of mechanistic evidence supporting the oral–gut–brain axis derives from animal models and in vitro studies; human data are largely observational and cross-sectional, precluding causal inference. Longitudinal interventional studies with neurological endpoints are scarce. Second, this review was conducted as a narrative review without a pre-registered protocol, formal risk-of-bias assessment, or independent dual-reviewer screening, which introduces the potential for selection bias and limits methodological reproducibility. Third, the heterogeneity of included studies, spanning different species, disease stages, biomarker assays, and microbiome profiling methods, limits direct comparability of findings. Fourth, the proposed multi-biomarker panel and integrated monitoring approach remain theoretical; no validated clinical instrument for simultaneous multi-compartment assessment of the oral–gut–brain axis currently exists. Fifth, several biomarkers discussed (particularly salivary BDNF, fecal SCFAs as neuroinflammatory proxies, and emerging miRNA signatures) lack clinical validation studies and should not be interpreted as ready for diagnostic application. Sixth, the therapeutic agents discussed in Section 7 are predominantly investigational; none are currently approved specifically for oral–gut–brain axis–directed therapy in neurodegenerative disease. These limitations underscore the need for rigorously designed, prospective, multi-center trials and call for caution in translating preclinical findings to clinical practice. Finally, it should be reiterated that this work is consistently described throughout the manuscript as a narrative review with structured (but non-systematic) elements. The use of the PRISMA 2020 checklist and flow diagram serves exclusively as a transparency tool and does not constitute a systematic review according to PRISMA methodological standards.

## 10. Conclusions

The evidence reviewed in this article supports the plausibility of a functionally integrated oral–gut–brain axis, in which periodontal dysbiosis may initiate a cascade of inflammatory events with repercussions extending from the oral cavity through the gastrointestinal tract to the CNS. This axis is a compelling conceptual framework supported by a convergent body of evidence from molecular biology, microbiology, immunology, neuroscience, and clinical epidemiology, though the strength of evidence varies substantially across its components and causal relationships in humans remain to be established.

The mechanistic pathways connecting these three compartments are biologically plausible and increasingly characterized in preclinical systems. Bacterial translocation from the oral cavity to the gut, disruption of intestinal barrier integrity, alteration of SCFA metabolism, metabolic endotoxemia, BBB compromise, and microglial activation represent a proposed pathogenic continuum documented in animal models and partially supported by human observational studies. These interconnected mechanisms offer a biological framework for understanding the epidemiological associations between periodontitis and cardiovascular, metabolic, and neurodegenerative diseases; however, prospective human data establishing causal relationships are still needed.

Salivary and intestinal biomarkers offer a potential window into this axis. Clinically validated markers (MMP-8/aMMP-8, IL-1β, IL-6 in saliva; calprotectin in feces) can non-invasively reflect periodontal and intestinal inflammatory burden. Emerging markers (salivary BDNF, fecal SCFAs, and miRNA signatures) require further prospective validation before clinical application. The development of integrated multi-biomarker panels, enabled by advances in point-of-care diagnostics and digital health technologies, could ultimately transform these markers from research tools into clinical decision support instruments, but this goal remains aspirational at present.

Pharmacological opportunities targeting the oral–gut–brain axis are diverse and scientifically promising, but most remain investigational. From established interventions (periodontal therapy, statins, sub-antimicrobial dose doxycycline) to emerging modalities (GLP-1 agonists, NLRP3 inflammasome blockers, specialized pro-resolving mediators, probiotics, and FMT), a rich preclinical landscape awaits rigorous clinical evaluation. No intervention currently has proven efficacy for neurodegeneration prevention through axis-directed mechanisms. The priority challenge lies in designing and executing rigorously controlled clinical trials that can establish efficacy, safety, and cost-effectiveness for these approaches, particularly for cognitive and neurological endpoints.

As a final methodological note, this work is presented as a narrative review with structured (but non-systematic) elements. The PRISMA-style reporting included as Supplementary Material is intended exclusively as a transparency tool and does not transform this review into a systematic review.

In conclusion, the management of periodontal, cardiometabolic, and neurodegenerative diseases can no longer be approached in disciplinary silos. The oral–gut–brain axis mandates an integrated, interprofessional model of care in which dental practitioners, physicians, pharmacists, and allied health professionals collaborate to address the systemic implications of oral health. Future research should prioritize longitudinal human studies establishing causality, multi-omics approaches for personalized risk profiling, and clinical trials of axis-targeted interventions, a research agenda that holds the potential to transform both preventive and therapeutic paradigms in chronic disease management.

## Figures and Tables

**Table 1:** Summary of salivary and intestinal biomarkers for monitoring the oral–gut–brain axis.

Biomarker	Specimen	Compartment Assessed	Biological Role	Clinical Utility	References
MMP-8/aMMP-8	Saliva	Oral	Collagenase-2; degrades type I collagen in the periodontal ligament	Periodontal disease activity monitoring; POC chair-side test available (PerioSafe^®^); associated with metabolic syndrome parameters	[116,118,119,120,121,122]
IL-1β	Saliva	Oral/Systemic	Key pro-inflammatory cytokine; principal mediator of osteoclast-mediated bone resorption	Periodontal severity indicator; correlates with probing depth, CAL, and BOP; reflects systemic inflammatory burden	[4,113,117]
IL-6	Saliva/Serum	Oral/Systemic	Pleiotropic cytokine; induces hepatic CRP synthesis; activates acute-phase response	Periodontal and cardiovascular risk indicator; elevated in periodontitis and correlates with serum CRP levels	[4,5,113]
CRP	Saliva/Serum	Systemic	Acute-phase protein; pentraxin synthesized by hepatocytes in response to IL-6	Cardiovascular risk screening; non-invasive monitoring potential; lab-on-a-chip platforms under development	[4,113,115]
BDNF	Saliva	Neurological	Neurotrophin essential for neuronal survival, synaptic plasticity, and cognitive function	Potential bridge biomarker linking oral inflammation to neurodegenerative risk; reduced in periodontitis patients	[62,123]
miR-146a/miR-155	Saliva	Oral/Systemic	Non-coding RNAs modulating NF-κB signaling and inflammatory gene expression	Emerging biomarkers of inflammatory regulation; differentially expressed in periodontitis; liquid biopsy potential	[114,124]
SCFAs (butyrate, acetate, propionate)	Feces	Intestinal/Neurological	Primary colonocyte energy source; HDAC inhibitor; maintains BBB integrity; promotes Treg differentiation	Intestinal dysbiosis indicator; neuroprotective signal; reduced butyrate correlates with barrier dysfunction and neuroinflammation	[62,63,64]
Calprotectin	Feces	Intestinal	Neutrophil-derived calcium-binding protein; marker of mucosal neutrophilic infiltration	Established IBD diagnostic marker; indicator of gut inflammation from ectopic oral pathogen colonization	[57,58]
Zonulin	Feces/Serum	Intestinal	Tight junction modulator; regulates paracellular permeability through reversible TJ disassembly	Intestinal barrier integrity (“leaky gut”) marker; associated with metabolic syndrome, autoimmune and neurological conditions	[58]

**Table 2:** Pharmacological and therapeutic strategies targeting the oral–gut–brain axis.

Intervention	Target Compartment	Mechanism of Action	Evidence Level	Key Findings	References
Non-surgical periodontal therapy (SRP)	Oral → Systemic	Mechanical removal of subgingival biofilm; reduction in local and systemic inflammatory burden	RCTs; meta-analyses	Reduces serum CRP, IL-6, TNF-α; improves HbA1c in T2DM; reduces blood pressure in hypertensive patients	[4,48,49,126,127]
Statins (HMG-CoA reductase inhibitors)	Systemic/CNS	Inhibit isoprenoid synthesis; reduce NF-κB activation; suppress pro-inflammatory cytokine production	Preclinical; observational	Anti-inflammatory and neuroprotective effects; reduce microglial activation and Aβ production in animal models	[4,92,129]
GLP-1 receptor agonists (semaglutide, liraglutide, exenatide)	Systemic/CNS	Reduce neuroinflammation, oxidative stress and neuronal apoptosis	Phase II/III trials	Neuroprotective in AD and PD animal models; clinical trials underway with promising preliminary results	[130,131,155,156]
Specialized pro-resolving mediators (SPMs: resolvins, protectins, maresins)	Multi-compartment	Actively promote inflammation resolution rather than suppression; endogenous lipid mediators	Preclinical	Reduce alveolar bone loss; promote tissue regeneration; attenuate systemic inflammatory responses	[132,133,134]
Gingipain inhibitors (COR388/atuzaginstat)	Oral/CNS	Block *P. gingivalis* cysteine protease activity; reduce bacterial virulence	Phase II/III (discontinued)	Reduce Aβ production and neuroinflammation in preclinical models; hepatotoxicity concerns in trials	[32,131,135]
Oral probiotics (*L. reuteri*, *L. rhamnosus*, *Bifidobacterium* spp.)	Oral	Competitive exclusion of pathogens; bacteriocin production; local immunomodulation	RCTs; meta-analyses	Adjunct to SRP; reduce PPD, CAL, and salivary pathogen load	[136,137,138]
Intestinal probiotics/prebiotics (FOS, GOS, resistant starch)	Intestinal	Selective stimulation of butyrate-producing commensals; enhance SCFA production; support barrier integrity	RCTs	Improve fecal SCFA profiles; restore Firmicutes/Bacteroidetes ratio; support intestinal barrier function	[63,139,140,141,142]
Fecal microbiota transplantation (FMT)	Intestinal → CNS	Restore microbiome diversity; modulate gut–brain signaling through normalized metabolite production	Preclinical; case reports	Reduced microglial activation and dopaminergic neurodegeneration in PD models; cognitive improvement in AD case report	[143,144,157,158]
Sub-antimicrobial dose doxycycline (SDD, Periostat^®^)	Multi-compartment	MMP inhibition; anti-inflammatory without antibiotic selective pressure at 20 mg BID	FDA-approved (periodontitis)	Reduces collagenase activity; long-term anti-inflammatory potential along the axis	[118,145]
NLRP3 inflammasome inhibitors (MCC950 and analogs)	Multi-compartment	Block caspase-1 activation; prevent IL-1β/IL-18 maturation and release	Preclinical	Efficacy demonstrated in preclinical models of periodontitis, IBD, and AD simultaneously	[59,147,148,150]

Symbols and abbreviations: → leads to/results in. Aβ, amyloid-beta; AD, Alzheimer’s disease; BBB, blood–brain barrier; FDA, U.S. Food and Drug Administration; IBD, inflammatory bowel disease; IDO1, indoleamine 2,3-dioxygenase 1; IL, interleukin; KAT, kynurenine aminotransferase; LPS, lipopolysaccharide; MMP, matrix metalloproteinase; NLRP3, NOD-like receptor family pyrin domain containing 3; SDD, sub-antimicrobial dose doxycycline; SRP, scaling and root planing.

## Data Availability

No new data were created or analyzed in this study. Data sharing is not applicable to this article.

## Supplementary Materials

The following supporting information can be downloaded at: https://www.mdpi.com/article/10.3390/dj14050289/s1. Table S1: PRISMA 2020 Checklist—completed reporting checklist documenting how each PRISMA 2020 item has been addressed in this manuscript, applied as a transparency-reporting tool given the narrative (non-systematic) nature of the review. Adapted from Page et al. [33].

## Abbreviations

The following abbreviations are used in this manuscript:

Abbreviation	Full Term
Aβ	Amyloid-beta
AD	Alzheimer’s disease
BBB	Blood–brain barrier
CNS	Central nervous system
CRP	C-reactive protein
FMT	Fecal microbiota transplantation
IL-1β	Interleukin-1 beta
IL-6	Interleukin-6
IL-8	Interleukin-8
IL-10	Interleukin-10
IL-18	Interleukin-18
LPS	Lipopolysaccharide
MMP-2	Matrix metalloproteinase-2
MMP-8	Matrix metalloproteinase-8 (collagenase-2)
MMP-9	Matrix metalloproteinase-9
NADPH oxidase	Nicotinamide adenine dinucleotide phosphate oxidase
NF-κB	Nuclear factor kappa B
NLRP3	NLR family pyrin domain containing 3
NO	Nitric oxide
NOD-like receptors	Nucleotide-binding oligomerization domain-like receptors
PD	Parkinson’s disease
*P. gingivalis*	*Porphyromonas gingivalis*
T2DM	Type 2 diabetes mellitus
AhR	Aryl hydrocarbon receptor
APP	Amyloid precursor protein
BDNF	Brain-derived neurotrophic factor
BOP	Bleeding on probing
CAL	Clinical attachment loss
ENS	Enteric nervous system
FFAR2/FFAR3	Free fatty acid receptor 2/3 (GPR43/GPR41)
GLP-1	Glucagon-like peptide-1
GSK-3β	Glycogen synthase kinase-3 beta
HDAC	Histone deacetylase
MCT	Monocarboxylate transporter
MMSE	Mini-Mental State Examination
MoCA	Montreal Cognitive Assessment
MS	Multiple sclerosis
NVU	Neurovascular unit
OMV	Outer membrane vesicle
PAMP	Pathogen-associated molecular pattern
POC	Point-of-care
PPD	Probing pocket depth
PRR	Pattern recognition receptor
ROS	Reactive oxygen species
SASP	Senescence-associated secretory phenotype
SCFA	Short-chain fatty acid
SDD	Sub-antimicrobial dose doxycycline
SPM	Specialized pro-resolving mediator
TLR	Toll-like receptor
α-syn	Alpha-synuclein
