# Supplementary material for: The Oral–Gut–Brain Axis: From Periodontal Dysbiosis to Neuroinflammation—Mechanistic Pathways, Salivary and Intestinal Biomarkers, and Therapeutic Targets: A Narrative Review
Source: Dent J (Basel). 2026 May 11;14(5):289. doi: 10.3390/dj14050289 (PMC13205872; doi:10.3390/dj14050289)
Supplement: Supplementary file 1 [file dentistry-14-00289-s001.zip › dentistry-4271932-supplementary.pdf]

Supplementary Table S1: PRISMA 2020 Checklist

Reporting checklist adapted from: Page MJ, McKenzie JE, Bossuyt PM, Boutron I, Hoffmann TC, Mulrow CD, et al. The PRISMA 2020 statement: an updated guideline for reporting systematic reviews. ***BMJ*** *2021;372:n71. https://doi.org/10.1136/bmj.n71*

This checklist documents how the PRISMA 2020 reporting items have been addressed in this manuscript. The PRISMA 2020 framework was used solely as a transparency-reporting tool; this work is a narrative review with structured (non-systematic) elements and does not fulfill the methodological requirements of a full systematic review. Items marked with a shaded background indicate sub-items that are not formally applicable given the narrative methodology, with rationale provided in the corresponding cell.

Section and Topic	Item #	Checklist Item	Location Where Item Is Reported
**TITLE**			
**Title**	1	Identify the report as a systematic review, meta-analysis, or both.	Title page: identified as a Narrative Review (with structured/non-systematic elements described in Methods).
**ABSTRACT**			
**Abstract**	2	See the PRISMA 2020 for Abstracts checklist.	Abstract: structured with Background, Objective, Results, and Conclusions.
**INTRODUCTION**			
**Rationale**	3	Describe the rationale for the review in the context of existing knowledge.	Section 1.1–1.3: epidemiological context, inflammaging mechanism, evolution from bidirectional to three-dimensional model.
**Objectives**	4	Provide an explicit statement of the objective(s) or question(s) the review addresses.	Section 1.4: central hypothesis and four specific objectives explicitly stated.
**METHODS**			
**Eligibility criteria**	5	Specify the inclusion and exclusion criteria for the review and how studies were grouped for the syntheses.	Section 2.2: detailed inclusion criteria (study types) and exclusion criteria.
**Information sources**	6	Specify all databases, registers, websites, organisations, reference lists and other sources searched or consulted to identify studies.	Section 2.1: PubMed/MEDLINE, Scopus, Web of Science, Google Scholar; reference list searching.
**Search strategy**	7	Present the full search strategy for at least one database, including any filters and limits used.	Section 2.1: MeSH terms and free-text keywords listed; Boolean operators described; time period 2000–March 2026.

Section and Topic	Item #	Checklist Item	Location Where Item Is Reported
**Selection process**	8	Specify the methods used to decide whether a study met the inclusion criteria of the review.	Section 2.3: structured approach with title/abstract screening, full-text evaluation, and quality assessment.
**Data collection process**	9	Specify the methods used to collect data from reports.	Section 2.3: narrative synthesis approach; data extracted by domain (microbiology, immunology, neuroscience, gastroenterology, clinical dentistry).
**Data items**	10a	List and define all outcomes for which data were sought.	Not applicable in formal PICO format; narrative review synthesizes mechanistic pathways, biomarkers, and therapeutic targets.
**Data items**	10b	List and define all other variables for which data were sought.	Key variables include inflammatory mediators, microbiome composition, barrier integrity markers, and neuropathological endpoints (described thematically in Sections 3–7).
**Study risk of bias assessment**	11	Specify the methods used for assessing risk of bias of included studies.	Not formally applied (narrative review). Systematic reviews and meta-analyses were prioritized as high-level evidence sources (Section 2.3). Acknowledged as limitation in Section 9.
**Effect measures**	12	Specify for each outcome the effect measure(s) used in the synthesis or presentation of results.	Not applicable; no quantitative or meta-analytic synthesis performed. Narrative integration of qualitative and quantitative findings.
**Synthesis methods**	13a–13f	Describe the processes used to decide which studies were eligible, tabulate data, assess risk of bias, and synthesize results.	Section 2.3: narrative review with structured (non-systematic) elements; thematic synthesis across domains. Formal sub-items 13a–13f for systematic synthesis are not applicable.
**Reporting bias assessment**	14	Describe any methods used to assess risk of bias due to missing results in a synthesis.	Not formally assessed; acknowledged as limitation in Section 9.
**Certainty assessment**	15	Describe any methods used to assess certainty in the body of evidence for an outcome.	Formal certainty rating (e.g., GRADE) not applied; evidence hierarchy discussed qualitatively (RCTs and meta-analyses prioritized).
**RESULTS**			

Section and Topic	Item #	Checklist Item	Location Where Item Is Reported
**Study selection**	16a	Describe the results of the search and selection process, ideally with a flow diagram.	Section 2.3 and Figure 2 (PRISMA 2020 flow diagram): 525 records identified (480 from database searching + 45 from citation tracking), 159 studies included after structured screening and eligibility assessment.
**Study selection**	16b	Cite studies that appeared to meet inclusion criteria but were excluded, and explain why.	Exclusion categories described in Figure 2 (PRISMA flow diagram). Individual study-level exclusion list not maintained, consistent with narrative review methodology.
**Study characteristics**	17	Cite each included study and present its characteristics.	The 159 studies synthesized in this review are cited throughout the manuscript; their characteristics are described in context within each thematic section. The reference list contains 160 entries: the 159 included studies plus the PRISMA 2020 statement [33], cited as a methodological reference.
**Risk of bias in studies**	18	Present assessments of risk of bias for each included study or outcome.	Not formally presented; limitations of individual study designs are discussed where relevant in the text. Acknowledged as limitation in Section 9.
**Results of individual studies**	19	For all outcomes, present, for each study, data for each intervention group with effect estimates and confidence intervals.	Not applicable for narrative review; key findings summarized thematically with source citations.
**Results of syntheses**	20a–20d	Present results of all syntheses, including statistical measures and sensitivity analyses.	Thematic narrative synthesis presented in Sections 3–7; Tables 1–2 summarize biomarkers and therapeutic strategies. Quantitative synthesis (sub-items 20b–20d) not applicable.
**Reporting biases**	21	Present assessments of risk of bias due to missing results for each synthesis.	Not formally assessed; acknowledged as limitation in Section 9.
**Certainty of evidence**	22	Present assessments of certainty in the body of evidence for each outcome.	Discussed qualitatively; evidence levels noted (RCTs, meta-analyses, preclinical, observational). Formal GRADE assessment not applied.
**DISCUSSION**			

Section and Topic	Item #	Checklist Item	Location Where Item Is Reported
**Discussion**	23a	Provide a general interpretation of the results in the context of other evidence.	Section 10 (Conclusions): integrative interpretation of oral–gut–brain axis evidence.
**Discussion**	23b	Discuss any limitations of the evidence included in the review.	Section 9: knowledge gaps including causality, quantitative contribution, and standardization needs.
**Discussion**	23c	Discuss any limitations of the review processes used.	Section 9: acknowledged as narrative review with structured elements; no formal risk-of-bias or GRADE assessment; single-reviewer screening; PRISMA used as transparency tool only.
**Discussion**	23d	Discuss implications of the results for practice, policy, and future research.	Section 8: multi-omics integration, personalized medicine, emerging technologies, and proposed research priorities.
**OTHER INFORMATION**			
**Registration and protocol**	24a–24c	Provide registration information including register name and number, or state that the review was not registered. Indicate deviations from protocol.	This narrative review was not registered in PROSPERO. No formal protocol was published a priori. Stated explicitly in Section 2 (Methodology).
**Support**	25	Describe sources of financial or non-financial support for the review.	Funding statement: no external funding; article processing charges supported by "Dunărea de Jos" University of Galați, Romania.
**Competing interests**	26	Declare any competing interests of review authors.	Conflicts of Interest statement: the authors declare no conflicts of interest.
**Availability of data, code and other materials**	27	Report which of the following are publicly available: template data collection forms, data extracted from included studies, data used for analyses, analytic code, any other materials.	Data Availability statement: no new data were created or analyzed; data sharing not applicable to this article.

Note: *As this is a narrative review with structured (non-systematic) elements rather than a fully systematic review, certain PRISMA items (formal risk-of-bias assessment, effect measures, meta-analytic synthesis methods, GRADE certainty assessment) are reported as not formally applied. Rows highlighted in cream/orange indicate items that are not applicable or only partially applicable given this hybrid methodology, consistent with the approach described in Section 2 (Methodology). The PRISMA 2020 statement is cited in the main reference list as reference [33]; this single methodological reference is the reason the reference list contains 160 entries while the PRISMA flow diagram (Figure 2) reports 159 studies included through the literature search.*
